# Circadian Clocks in Mouse and Human CD4+ T Cells

**DOI:** 10.1371/journal.pone.0029801

**Published:** 2011-12-28

**Authors:** Thomas Bollinger, Anton Leutz, Alexei Leliavski, Ludmila Skrum, Judit Kovac, Luigi Bonacina, Christian Benedict, Tanja Lange, Jürgen Westermann, Henrik Oster, Werner Solbach

**Affiliations:** 1 Institute of Medical Microbiology and Hygiene, University of Lübeck, Lübeck, Germany; 2 Department of Neuroendocrinology, University of Lübeck, Lübeck, Germany; 3 Institute of Anatomy, University of Lübeck, Lübeck, Germany; 4 Max Planck Institute of Biophysical Chemistry, Göttingen, Germany; 5 GAP – Biophotonics, University of Geneva, Geneva, Switzerland; 6 Department of Molecular Biology, University of Geneva, Sciences III, Geneva, Switzerland; McGill University Health Center, Canada

## Abstract

Though it has been shown that immunological functions of CD4+ T cells are time of day-dependent, the underlying molecular mechanisms remain largely obscure. To address the question whether T cells themselves harbor a functional clock driving circadian rhythms of immune function, we analyzed clock gene expression by qPCR in unstimulated CD4+ T cells and immune responses of PMA/ionomycin stimulated CD4+ T cells by FACS analysis purified from blood of healthy subjects at different time points throughout the day. Molecular clock as well as immune function was further analyzed in unstimulated T cells which were cultured in serum-free medium with circadian clock reporter systems. We found robust rhythms of clock gene expression as well as, after stimulation, IL-2, IL-4, IFN-γ production and CD40L expression in freshly isolated CD4+ T cells. Further analysis of IFN-γ and CD40L in cultivated T cells revealed that these parameters remain rhythmic *in vitro*. Moreover, circadian luciferase reporter activity in CD4+ T cells and in thymic sections from *PER2::LUCIFERASE* reporter mice suggest that endogenous T cell clock rhythms are self-sustained under constant culture conditions. Microarray analysis of stimulated CD4+ T cell cultures revealed regulation of the NF-κB pathway as a candidate mechanism mediating circadian immune responses. Collectively, these data demonstrate for the first time that CD4+ T cell responses are regulated by an intrinsic cellular circadian oscillator capable of driving rhythmic CD4+ T cell immune responses.

## Introduction

24-h rhythms of behavior (e.g. sleep/wake cycle) and physiology (e.g. hormone secretion, energy demands and immune responses) are the external manifestation of internal clocks that measure daytime [1]–[6]. In mammals these circadian clocks (from Latin *circa dies –* around a day) are organized in a hierarchical way. The hypothalamic suprachiasmatic nucleus (SCN) harbors a master circadian pacemaker which synchronizes peripheral clocks all over the body by various factors including hormones, the sympathetic nervous system (SNS), and body temperature rhythms [3], [7]. Disruption of circadian synchrony has been shown to be detrimental for metabolic homeostasis [3], [8]. The effect of circadian disruption on immune responses is almost unknown, but “jet lag” experiments in mice and data from clock gene deficient animals indicate strong effects of circadian disruption on innate immune responses [9], [10].

On the molecular level circadian oscillators consist of transcriptional/translational feedback loops involving a number of clock genes/proteins. The transcription factors aryl hydrocarbon receptor nuclear translocator-like (ARNTL or BMAL1) and circadian locomotor output cycles kaput (CLOCK) activate the period (*Per1-3*) and cryptochrome (*Cry1-2*) genes. After accumulation in the cytoplasm PER/CRY complexes relocate into the nucleus to inactivate CLOCK/BMAL1 transactivation, thereby down-regulating their own expression. This core feedback loop is stabilized by several ancillary loops including the genes for retinoic acid receptor-related orphan receptors alpha (*Rorα*), orphan nuclear receptor NR1D1 (*Nr1d1* or *Rev-erbα*), nuclear factor, interleukin 3 regulated (*Nfil3* or *E4bp4*), and D site of albumin promoter (albumin D-box) binding protein (*Dbp*). Circadian clocks have been described in many cell types including neurons, fibroblasts, hepatocytes, kidney and adrenal cells. Furthermore, the circadian clock is highly conserved throughout evolution underlining the outstanding importance of this mechanism for survival. Despite the fact that circadian symptoms of immunological disease are well known, e.g. in rheumatoid arthritis [11], only few studies have addressed the existence and function of circadian clocks in cells of the immune system, and until now this has only been shown for cells of the innate immune system and peripheral blood mononuclear cells (PBMCs) [12]–[15]. Rat natural killer (NK) cells harbor a circadian clock driving circadian rhythms of NK cell immune responses such as granzyme B and perforin production [12], [16]–[19]. In mouse peritoneal macrophages a cellular clock regulates the response to lipopolysaccharide (LPS) stimulation. Microarray analysis revealed several genes involved in LPS signaling and response pathways to be under circadian control [14], [20]. Furthermore, mortality after LPS-induced septic shock is regulated via IFN-γ, depending on the circadian time of LPS application, and this regulation is modulated by the clock gene *Per2* [10], [21]. On the other hand, it has been shown that cytokines can feed back on clock function by modulating the expression of clock genes in different tissues [22]. In summary, these data implicate a tight connection of the innate immune system and circadian clocks.

In contrast, little is known about the influence of circadian/diurnal time on the adaptive immune response so far. Two studies investigating the immunological response to hepatitis A and B vaccination found a substantial effect of the time of application and, thereby, antigen presentation in the lymph node [23], [24]. Furthermore, in arrhythmic *Cry1/Cry2* double deficient mice disease severity of rheumatoid arthritis is substantially exacerbated [25]. Whether this CD4+ T cell-dependent phenomenon is driven by rhythmic systemic factors (such as hormones) or by intrinsic cellular circadian oscillators in antigen presenting cells, T cells or B cells, is currently unknown. CD4+ T cells are key regulators of adaptive immune responses and we have previously shown that CD4+ T cell proliferation as well as cytokine production follows a circadian/diurnal rhythm [26], [27]. In this study, we investigated whether CD4+ T cells harbor an intrinsic timekeeper capable of regulating circadian T cell immune responses.

We demonstrate by mRNA expression analysis of freshly isolated as well as *in vitro* cultured CD4+ T cells, and by clock reporter systems, that these cells contain their own circadian clock. Freshly isolated CD4+ T cells showed a circadian rhythm of CD40L expression, IL-2, IL-4, and IFN-γ production after phorbol myristate acetate (PMA)/ionomycin stimulation. This rhythm of IFN-γ production and CD40L expression was sustained for at least 48 h in culture. Microarray and subsequent real-time quantitative (q)PCR analysis revealed the NFκB pathway as one of the possible mediator of the circadian T cell response. Collectively, our data suggest for the first time that intrinsic circadian clocks can regulate the circadian responsiveness of CD4+ T cells.

## Materials and Methods

### Ethical statement

This study was carried out in strict accordance with the recommendations in the Guide for the Care and Use of Laboratory Animals of the National Institutes of Health. Animal experiments were approved by the Office for Consumer Protection and Food Safety of the State of Lower Saxony, Germany (LAVES; ID 33.11.42502-04-095/07). All human subjects gave written consent and the protocol was approved by the ethic commission of the University of Lübeck.

### Circadian imaging of *PER2::LUCIFERASE* thymic slices

To investigate whether circadian rhythms in clock gene mRNA/protein expression can be observed in primary lymphoid organs we utilized *PER2::LUCIFERASE* reporter mice [28]. These mice express the PER2 protein as a fusion protein together with the firefly LUCIFERASE enzyme. In luciferin-containing media the amount of emitted photons directly reflects the concentration of PER2 in the cells. We isolated the thymus of male heterozygous *PER2::LUCIFERASE* mice, embedded one of the thymic lobes in 4% UltraPure low melting point agarose (Invitrogen) and prepared 200 µm thick sections on a vibratome (Campden Instruments, Loughborough, UK). One section was cultured at 37°C, 5% CO_2_ on a 0.4 µm Millicell-CM PTFE membrane (Millipore, Billerica, USA) in DMEM (Invitrogen) supplemented with luciferin (200 nM) for approximately 5 days. Luminescence was imaged with a LV200 imaging system (Olympus, Hamburg, Germany). A second section was frozen in liquid nitrogen; air dried and stored at −80°C. Specimens were fixed in chloroform (10 min), immediately transferred into acetone (10 min.), washed in PBS and then kept in 4% paraformaldehyde for 45 min. After washing they were stained with haemalaun for 10 min, washed and mounted.

### Subjects and procedure

Cells were collected from seven healthy non-smoking male subjects (age, 23.14±1.5 yrs; BMI, 23.6±0.77 kg/m^2^). An interview prior to the study assured that participants had a regular sleep–wake rhythm for at least 6 weeks before the experiments and were not on medication. Acute illness was excluded by physical examination and routine laboratory investigation. The week before the experiment, subjects were required to turn off lights for nocturnal sleep between 11 and 11:30 PM, to get up by 7 AM the next morning, and not to take any naps during the day. The presence of sleep disturbances was excluded by sleep monitoring in a separate adaptation night which also served to habituate subjects to the experimental setting and took place within a week before the subjects' first experimental session. The study was approved by the ethics committee of the University of Luebeck. All subjects gave written informed consent and were paid for participation.

Subjects arrived at the laboratory at 4:30 PM on the baseline day. First, they were prepared for blood sampling, polysomnographic recordings, and continuous measurement of core body temperature, heart rate, and trunk movements (see below). Experimental protocols started at 6 PM on the baseline day and lasted for 24 h (i.e., 6 PM of the following day). Ambient temperature was held constant (23°C). Lights were turned off at 11 PM and subjects were wakened between 6:30 and 7 AM when entering light non-rapid eye movement (NREM) sleep (NREM sleep stages 1 or 2). All subjects received standardized meals throughout the experiment.

### Blood sampling and CD4+ T cell isolation

Blood sampling was performed at 3 h intervals from an adjacent room via long thin tubes without disturbing the subject's sleep. For blood sampling an intravenous catheter was placed in the *vena cephalica*. To prevent clotting, 500 ml of 0.9% saline solution (without anticoagulant) were infused throughout the 24 h experimental period.

Blood samples were processed immediately after sampling. CD4+ T cells were isolated using “Whole blood CD4+ MicroBeads” (Miltenyi Biotec, Bergisch Gladbach, Germany) and the AutoMacs magnetic separator (Miltenyi Biotec) following the manufacturer's instructions. Isolated CD4+ T cells were split into two fractions used for either mRNA analysis or for functional assays (see below). Purity of the isolated CD4+ T cells was controlled by staining 2×10^4^ isolated cells with αCD4 monoclonal antibody (mAb) or an isotype control and analysis by flow cytometry (FACS; FacsCalibur, BD Biosciences, Heidelberg, Germany).

### Analysis of CD4+ T cell responses ex vivo

Freshly isolated CD4+ T cells were immediately resuspended in quantities of 2×10^5^ cells in 200 µl X-VIVO15 (Lonza, Basel, Switzerland) containing 5 ng/ml PMA (Sigma-Aldrich, Taufkirchen, Germany) and 500 ng/ml ionomycin (Sigma-Aldrich) and incubated for 6 h at 37°C, 5% CO_2_. After one hour, 6 nmol monensin (Sigma-Aldrich) was added. Subsequently, cells were fixed utilizing the Inside Staining Kit (Miltenyi Biotec) and analyzed by flow cytometry for the expression of CD4, CD40Ligand (CD40L, BD Biosciences), interleukin 2 (IL-2), interleukin 4 (IL-4), interleukin 17 (IL-17), and interferon gamma (IFN-γ, Miltenyi Biotec). Flow cytometry was performed using the FacsCalibur (BD Biosciences) and the FloJo Software (Tree Star, Ashland, USA).

### mRNA expression analysis ex vivo

2×10^5^ CD4+ T cells were lysed immediately after isolation and total RNA extracted using the NucleoSpin RNA 2 Kit (Machery-Nagel, Düren, Germany) and stored at −80°C. The isolated RNA was transcribed into cDNA utilizing the Transcriptor Reverse Transcriptase Kit (Roche, Basel, Switzerland). qPCR was performed applying the Light Cycler Taq Man Master Kit and the Universal Probe Library (Roche) for the following clock genes: *Bmal1*, *Clock*, *Per2*, *Per3*, *Cry1*, *Cry2*, *Rorα*, *Rev-erbα*, *E4bp4*, and *Dbp*. To investigate whether T helper cell immune responses are regulated in a circadian fashion we analyzed the mRNA expression of *IL-2*, *IFN-γ*, *CD40L* and nuclear factor of kappa light polypeptide gene enhancer in B-cells inhibitor, alpha (*IκBα*). Expression for all target genes was normalized to the housekeeping genes phophoribosyl-transferase (*HPRT*), porphobilinogen deaminase (*PBGD*), glucose-6-phosphate dehydrogenase (*G6PDH*), and beta-2-microglobulin (*B2M*). The percent of mean of a target gene was calculated for each reference gene. Then the average of all four percent of mean values was calculated. Each value was measured in three independent runs on the LightCycler 1.2 (Roche). Primer pairs and probe library ID for each transcript are depicted in Table. S1.

### Circadian gene expression in CD4+ T cells in vitro

CD4+ T cells were isolated at the beginning of the experimental session (6 PM) and cultured in quantities of 2×10^5^ cells in 200 µl X-VIVO15 at 37°C, 5% CO_2_. Every 3 h over a 24 h period one sample was lysed for RNA isolation and transcription of cDNA (see above) to quantify the expression of the clock genes *Bmal1*, *Clock*, *Cry1*, *Cry2*, *Per2*, *Per3*, *Rorα*, *Rev-erbα*, *Dbp*, *E4bp4* and of the immune genes *IFN-γ* and *CD40L*.

### CD4+ T cells from *PER2::LUCIFERASE* reporter mice

CD4+ T cells were isolated from *PER2::LUCIFERASE* reporter mice by negative Macs isolation (Miltenyi Biotec) and cultured in DMEM (Gibco, USA)+10%FCS+0.5 nM luciferin (Invitrogen, Karlsruhe, Germany)+0.5 ng/ml PMA. Luminescence was recorded with the LumiCycle (Actimetrics, Wilmette, USA) and data were analyzed using the LumiCycle Analysis Software (Actimetrics, Wilmette, USA).

### Circadian immune responses of CD4+ T cells in vitro

In order to analyze the circadian production of IFN-γ and CD40L in polyclonally stimulated CD4+ T cells *in vitro*, CD4+ T cells were isolated at 10 AM and quantities of 2×10^5^ cells were cultured in 200 µl X-VIVO15 at 37°C, 5% CO_2_. Every 6 h over a 48 h period one fraction of cells was stimulated with PMA/ionomycin for 6 h and CD40L as well as IFN-γ production were measured as described above. To assure the viability of the cultured cells we measured the amount of non viable cells after 0 h, 24 h and 48 h in culture applying propidium iodid staining (Invitrogen, Darmstadt, Germany) and subsequent FACS analysis as well as tryphan blue (Sigma, München, Germany) staining and microscopic analysis.

### Microarray analysis of circadian immune responses of CD4+ T cells in vitro

To characterize the transcriptional events underlying the activation pattern of the *in vitro* sustained circadian rhythm of IFN-γ production and CD40L expression in CD4+ T cells after 6 h stimulation with PMA/ionomycin, we performed cRNA hybridization to whole genome microarrays. CD4+ T cells were isolated and cultured as described above. Every 6 h over a 48 h period, one fraction of cells was stimulated with PMA/ionomycin for 3 h, shock frozen in liquid nitrogen and stored at −80°C. Total RNA was isolated using Trizol reagent (Invitrogen), biotin-labeled and hybridized to Affymetrix Human 1.0 ST Arrays (Affymetrix, Santa Clara, California, USA) using standard protocols [29]. All microarray data are MIAME compliant and have been deposited at GEO (accession number: GEO29583). Statistical analysis described below.

### Sleep, hormones, core body temperature, and trunk movement

Sleep stages were determined off-line from polysomnographic recordings and were categorized as normal according to standard criteria [30]. To ensure that the analyzed subjects show normal hormone, temperature, and activity rhythms we monitored core body temperature, heart rate, and serum/plasma levels of cortisol, melatonin, prolactin, and adrenalin as described (Benedict et al., in revision). The measures of the controls revealed normal circadian rhythm of all subjects and are shown in Figure S1 and Table S2.

### Statistical analyses

Rhythm peak time analyses were performed using Prism software (GraphPad). A sine wave (*y* = BaseLine+Amplitude sin (frequency *x*+PhaseShift)) with a fixed period of 24 h was fitted to the data. Circadian rhythm of the percent of mean of all parameters was analyzed by Cosinor analysis [31]. Microarray data analysis was performed as follows: (i) between-array normalization, (ii) PCA-analysis, (iii) fitting the data to a linear model, and (iv) detection of differential gene expression. Quantile-normalization was applied to the log2-transformed intensity values as a method for between-array normalization to ensure that the intensities had similar distributions across all arrays [32]. PCA was performed using the *princomp* function of the R software package (version 2.10; available from: www.r-project.org). To identify periodic genes the array data of the first and the third time point were merged to one group and compared to the intermediate time point using a simple ANOVA. Genes with a p-value below 0.01 and a log2-fold change >0.2 were classified as “periodic”.

For the analysis of IFN-γ and CD40L over 48 h *in vitro* we used a different fitting procedure since the analysis of two complete circadian cycles allowed for a more precise fitting. The data from the four patients were first used for calculating the mean and standard deviation at each time point for the different reporters, followed by a least square fitting procedure using the following formula:
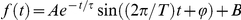
with *A* and *T* depicting amplitude and period, τrepresenting a time constant accounting for the decay of the oscillations' amplitude, φthe initial phase, and *B* the signal offset. During the automatic fitting process (Igor PRO, Wavemetrics) the data were weighted by their respective standard deviations. The goodness of the fit was then tested by calculating the reduced χ^2^ value assuming 4 degrees of freedom (9 time points minus the 5 parameters *A*, τ, *T*, ϕ, *B* calculated from the data) and compared to the probability *P* of the χ^2^ distribution.

## Results

### Circadian imaging of *PER2::LUCIFERASE* thymic slices

**Figure 1:**
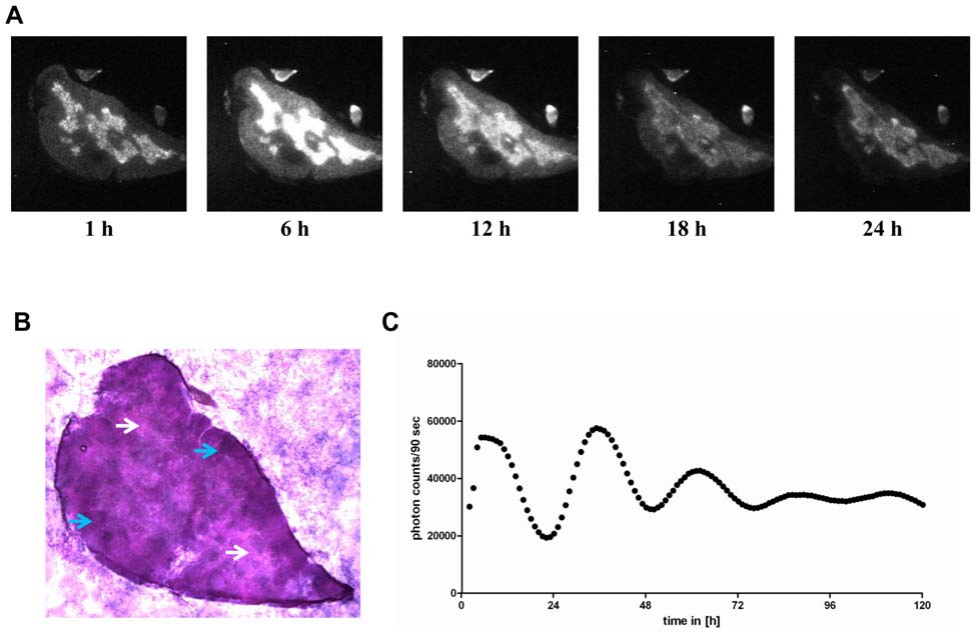
Bioluminescence microscopy of *PER2::LUCIFERASE* thymic sections. Male *PER2::LUCIFERASE* reporter mice were sacrificed and the thymus lobe was sliced. One section was put into medium (DMEM) supplemented with luciferin and light emission was continuously imaged (10-fold magnification applying the Olympus LV200) over approximately five days at 37°C. A) Depicted are microscopic images in six hour intervals of the first 24 h (the complete microscopic video is in the supplemental material). B) A section of the in A analyzed thymus lobe was stained with haemalaun/eosin to visualize cortex and medulla of the thymus lobe (2.5-fold magnification). White arrows show the medulla (light purple regions) and blue arrows show the cortex (dark purple regions). C) Shows the quantified amount of light emission by the thymic slice over the whole recording time. Shown are data of one out of two experiments (period length = 26 h).

As a “prove of principal experiment” we first investigated whether T cells have a functional circadian clock in a mouse reporter system of the circadian clock. Therefore, we established an *ex vivo* culture of a primary lymphoid organ. Thymic slices from *PER2::LUCIFERASE* reporter mice were cultured and imaged for light emission. In this reporter mouse Period2 (an essential protein of the circadian clock) is expressed as a fusion protein with the firefly luciferase. Hence, if thymic cells, which are mostly T cells, have a functional circadian clock we should observe circadian light emission in the culture. Robust rhythms of luciferase activity were recorded for more than four days in culture (see Fig. 1 and the supplemental video (Video S1), but by this approach we cannot exclude that thymic, e.g., stroma cells are responsible for the observed circadian rhythm. The haemalaun-eosin staining of the thymic section showed that the rhythmic luciferase activity is almost exclusively detected in the medulla (Fig. 1).

### Circadian immune responses and clock gene expression in CD4+ T cells ex vivo

**Figure 2:**
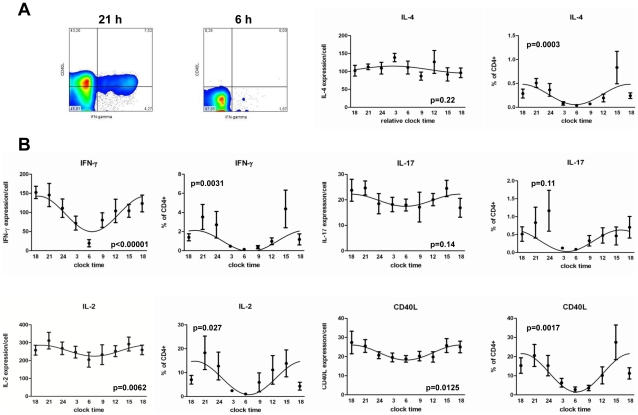
Circadian T cell activity ex vivo. Blood was sampled from seven healthy young males in three hour intervals starting at 6 PM over a 24 h period. CD4+ T cells were isolated from whole blood by MACS technology and the purified CD4+ T cells (mean purity: 94.99%±0.5%) were stimulated six hours with PMA/ionomycin. Cells were then fixed and CD40L, IL-2, IL-4, IL-17, and IFN-γ expression was analyzed by FACS. A) Shows two FACS plots of one donor at the peak and trough of IFN-γ production (time points as indicated). B) The graphs depict the GeoMean data (expression/cell), the percent of CD40L+ cytokine+ CD4+ T cells and the percent of CD40L+ CD4+ T cells as indicated. The p-values depicted in each graph were calculated by Cosinor analysis (Table. S2).

**Figure 3:**
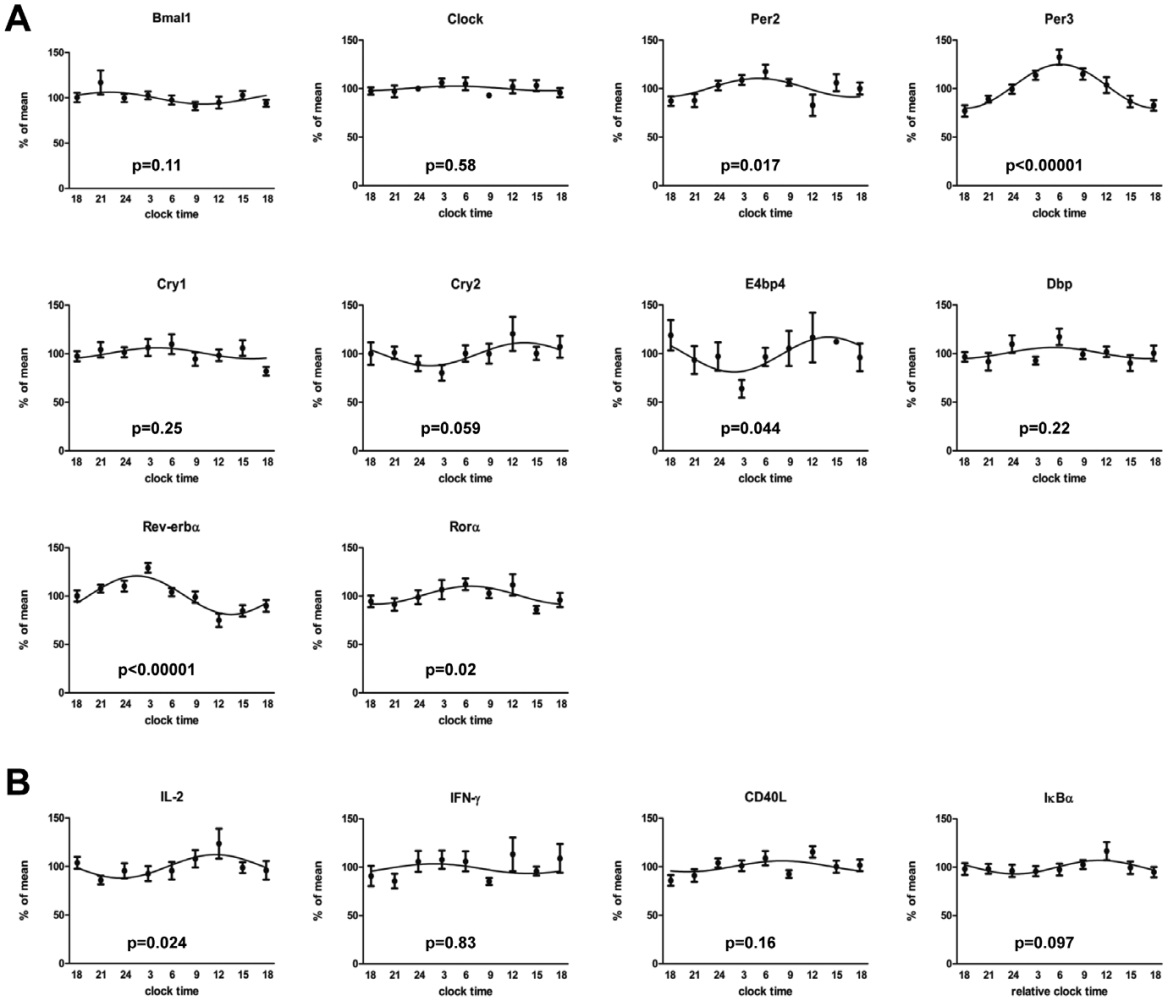
Circadian clock gene expression in purified CD4+ T cells ex vivo. Blood was sampled from seven healthy young males in three hours intervals starting at 6 PM over a 24 h period. CD4+ T cells were isolated from whole blood by MACS technology and the purified CD4+ T cells (mean purity: 94.99%±0.5%) were lysed, RNA was isolated, and the mRNA of ten clock genes was analyzed by qPCR. Depicted are the mRNA levels of clock genes (A) and immune genes (B) relative to the reference genes *B2M*, *HPRT*, *PBGD*, and *G6PDH*. The p-values depicted in each graph were calculated by Cosinor analysis (Table. S2).

After finding circadian luciferase expression in thymus slices from *PER2::LUCIFERASE* reporter mice we wanted to investigate circadian T cell immune responses. It was previously described that T cell activity in the presence of other immune cells (e.g. antigen presenting cells (APCs)) follows a circadian/diurnal rhythm [6], [14]. The question of whether the circadian immune response of T cells is due to a circadian rhythm in T cells themselves or due to external time cues, e.g. from APCs, is currently unresolved. To test this, we polyclonally stimulated highly purified human CD4+ T cells sampled from healthy young males at 3 h intervals over a 24 h period. In these cells we found circadian rhythms in the production of IFN-γ, IL-2, IL-4, and CD40L with peaks in the late evening and troughs around 6 AM. No obvious rhythm was detected for IL-17 (Fig. 2). One possible explanation for the observed circadian immune response of CD4+ T cells would be the presence of an intrinsic circadian clock in CD4+ T cells, even though differences in the composition of CD4+ T cells in terms of the percentage of naive, regulatory, effector or memory T cells as well as the influence of systemic cues as light/dark cycle or rhythmic hormone secretion (e.g. cortisol) cannot be excluded by this approach. To assess circadian clock gene expression in freshly isolated and unstimulated T cells we quantified mRNA expression levels of ten key clock genes on the same samples [8]. We found significant circadian rhythms of expression for *E4bp4*, *Per2*, *Per3*, *Rev-erbα*, and *Rorα* in all subjects (Fig. 3A, Table. S2), whereas there was a trend for *Cry2*. No significant circadian rhythm was detected for *IFN-γ* transcription in non-stimulated CD4+ T cells, whereas there was a trend or significant circadian rhythm for *IκBα* and *IL-2* mRNA expression, respectively (Fig. 3B, Table. S2).

### Circadian immune gene and clock gene expression in CD4+ T cells in vitro

**Figure 4:**
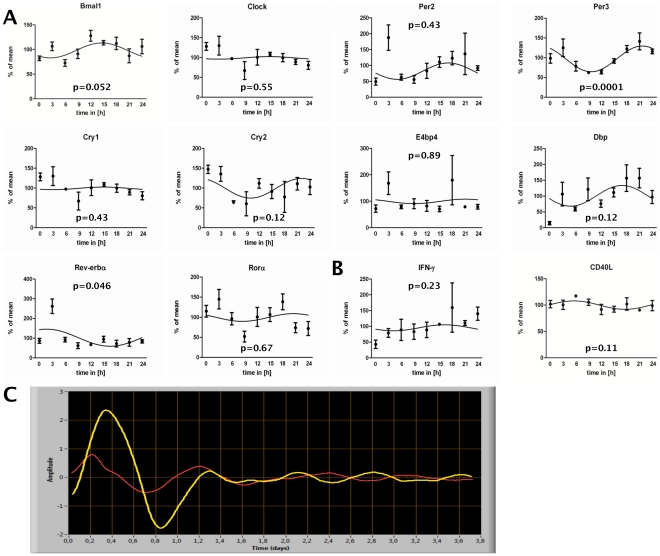
Circadian clock gene expression in in vitro cultured CD4+ T cells and *PER2::Luciferase* CD4+ reporter T cells. Blood was sampled from three healthy young males at 6 PM. CD4+ T cells were isolated from whole blood by MACS technology and the purified CD4+ T cells (mean purity: 96.4%±1.7%) and subsequently cultured in serum free medium. Every three hours over a 24 h period cells were collected, lysed, RNA was isolated, and clock genes expression analyzed by quantitative RT-PCR. A+B) depict the mRNA expression of clock genes (A) and immune genes (B) relative to the reference genes *B2M* and *HPRT*. The x-axis reflects the time cells were in culture. C) CD4+ T cells (purity: spleen = 88%, thymus = 96.7%) were isolated from spleen (red line, period = 24 h) and thymus (yellow line, period = 26.5 h) of *Per-Luc* reporter mice and cultured in the presence of 0.5 ng/ml PMA. Data shown are from one of five (spleen derived CD4+ T cells) and one out of two (thymus derived CD4+ T cells) independent experiments.

To test if the above shown circadian rhythm of clock genes in freshly isolated CD4+ T cells is sustained *in vitro*, we isolated CD4+ T cells and cultured them for a period of 24 h. This approach also rules out that circadian differences in T cell composition are the cause of circadian variation in clock gene expression in CD4+ T cells. Therefore, every 3 h cells were taken out of culture and clock gene expression was analyzed by qPCR. As shown in Fig. 4A, we found a trend or significant circadian gene expression rhythms for *Bmal1*, *Per3*, and *Rev-erbα* (Table. S2). The rhythm of *Cry2* and *Dbp* was donor-dependent. A donor-dependent circadian rhythm could also be detected for the expression of *IFN-γ* and *CD40L* in non-stimulated CD4+ T cells *in vitro* (Fig. 4B, Table. S2).

### CD4+ T cells from *PER2::LUCIFERASE* reporter mice

To establish a circadian *in vitro* reporter system we used freshly isolated splenic and thymic CD4+ T cells from *PER2::LUCIFERASE* reporter mice and could show that the luciferase activity followed a circadian rhythm (Fig. 4C). Since unstimulated mouse T cells do not survive for several days *in vitro* we stimulated the CD4+ T cells with a low dose (0.5 ng/mL) of PMA.

### Circadian immune responses of CD4+ T cells in vitro

**Figure 5:**
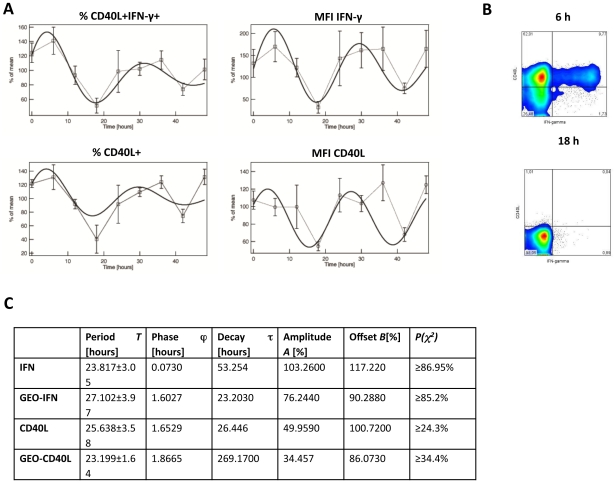
Circadian immune response of stimulated CD4+ T cells in vitro. Blood was sampled from four healthy young males at 10 AM. CD4+ T cells were isolated from whole blood by MACS technology and the purified CD4+ T cells (mean purity: 91.14%±0.82%) were subsequently cultured in serum-free medium and stimulated with PMA/ionomycin. A) Depicts the expression (GeoMean) as “percent of mean” of IFN-γ (mean = 207.74) and CD40L (mean = 54.56) per cell as well as the “percent of mean” of the percent of CD40L+CD4+(mean = 63.16%) and CD40L+IFN-γ+ CD4+ T cells (mean = 9.8%). The x-axis reflects the time cells were in culture. The statistical analyses of the fitting are shown in the table (C). The p-values calculated by Cosinor analysis are depicted in Table. S2. B) Shows two FACS plots of one donor at the peak and trough of IFN-γ production (time point as indicated).

Our data so far showed that CD4+ T cells display circadian rhythms of clock gene expression and that IFN-γ as well as CD40L production of freshly isolated and polyclonally stimulated CD4+ T cells follows a robust circadian rhythm. To test if this putative T cell clock is functional and drives the observed circadian rhythm in immune responses, we analyzed IFN-γ production and CD40L expression in *in vitro* cultured CD4+ T cells after stimulation. We chose IFN-γ and CD40L as markers for our *in vitro* approach since these markers had pronounced rhythms in our fresh *ex vivo* analyses. CD4+ T cells isolated in the morning were separated into aliquots and cultured *in vitro*. Every 6 h over a 48 h period three aliquots (on each time point) were polyclonally stimulated for 6 h (at 37°C, 5% CO2) and analyzed for IFN-γ and CD40L production. The rate of apoptotic/dead cells was 2.61±0.9 in freshly isolated CD4+ T cells, 5.2±0.95 after 24 h and 3.6±0.96 after 48 h in culture. As shown in Fig. 5 the rhythm of IFN-γ and CD40L production was sustained *in vitro*. We could detect at least two full cycles with a period length of approximately 24 h (Table. S2). However, the amplitude and is dampened in the second 24 h. Please note that the percent of CD40L and IFN-γ positive cells is higher than in the *ex vivo* stimulation. This might be explained by a longer isolation time and/or the fact that in this assay PMA/ionomycin were pre-diluted in medium before addition to the cells.

### Microarray analysis of circadian immune responses of CD4+ T cells in vitro

**Figure 6:**
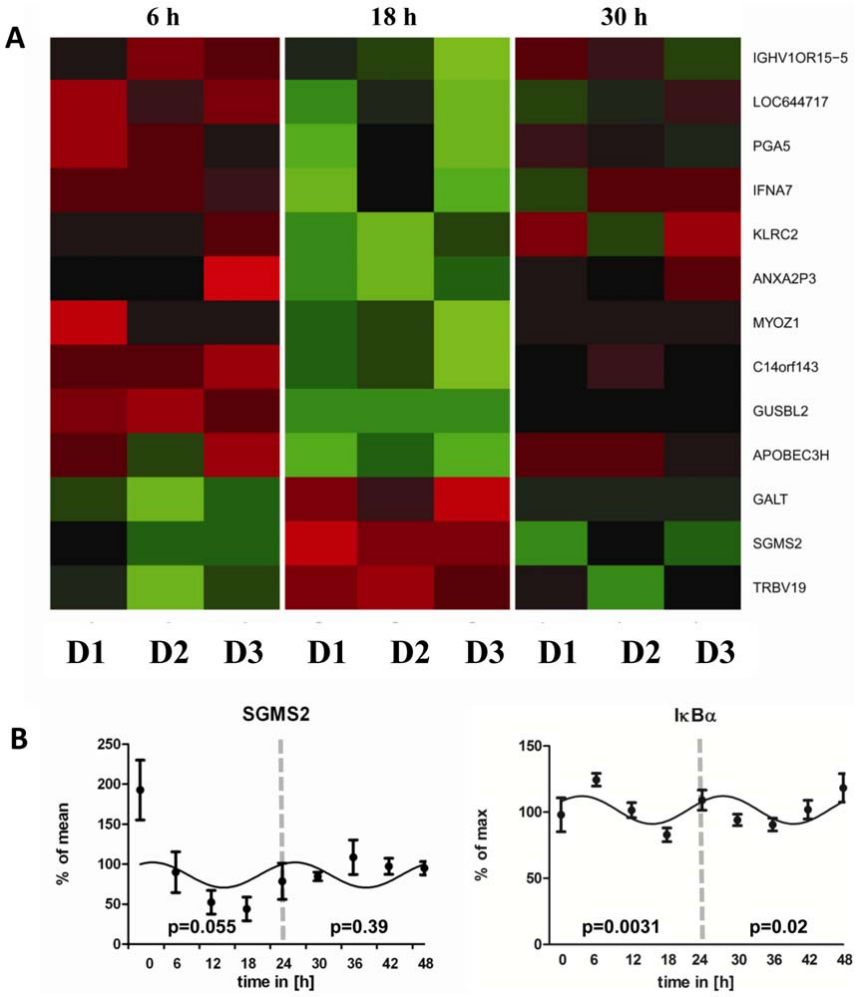
Circadian microarray and qPCR analysis of stimulated CD4+ T cells in vitro. Blood was sampled from three healthy young males at 10 AM. CD4+ T cells were isolated from whole blood by MACS technology. Aliquots of purified CD4+ T cells (mean purity: 90.94%±0.46%) were cultured up to 48 h. For microarray analysis aliquots were taken out in 6 h intervals and stimulated. Cells were stimulated 3 h with PMA/ionomycin. At the first and second peak as well as at the first trough of IFN-γ production cells were harvested for microarray analysis to identify candidate genes involved in the circadian regulation of IFN-γ production. A) Heat map of candidate genes identified by ANOVA. B) qPCR was performed from all time points (9 time points over 48 h) for *SGMS2* and *IκBα*. The p-values depicted in each graph were calculated by Cosinor analysis (Table. S2). The x-axis reflects the time cells were in culture. The dashed line splits the first and second 24 h period. P-values were separately calculated for the first and second 24 h and are depicted respectively.

After establishing an *in vitro* system for circadian immune responses in CD4+ T cells we wanted to identify the mechanistic link between clock gene expression rhythms and the circadian gating of IFN-γ and CD40L production. Here we choose our *in vitro* system in order to rule out that systemic cues account for differences in mRNA expression, therefore the expected differences must be driven by an internal circadian oscillator in CD4+ T cells. We performed microarray analysis of the above described *in vitro* cultured and stimulated CD4+ T cells. To identify candidate genes of the circadian regulation of T cell immune responses we analyzed stimulated CD4+ T cells from three different time points corresponding to the first maximum (6 h), first minimum (18 h), and second maximum (30 h) of IFN-γ and CD40L production. To account for the different kinetics in protein and mRNA expression we used CD4+ T cells which were polyclonally stimulated for 3 h. This microarray identified 13 significantly regulated candidate genes (Fig. 6A). Of particular interest was sphingomyelin synthase 2 (*SGMS2*), a known regulator of the activity of nuclear factor of kappa light polypeptide gene enhancer in B-cells inhibitor (NF-κB) [33]. We validated *SGMS2* mRNA rhythms by qPCR (Fig. 6B, Table. S2). In addition, we found rhythmic expression of nuclear factor of kappa light polypeptide gene enhancer in B-cells inhibitor, alpha (*IκBα*) mRNA in these cells by qPCR (Fig. 6B, Table. S2). NF-κB is a key regulator of *IκBα* transcription [34]. Thus, together these results suggest a circadian rhythm of the transcriptional activity of NF-κB, but we cannot exclude other mechanisms such as circadian variation in mRNA stability.

## Discussion

In this study, we investigated T helper cell activity and its regulation by the circadian clock. We showed that clock genes are rhythmically expressed in freshly isolated as well as in *in vitro* cultured primary human CD4+ T cells. Further, we established a reporter T cell assay utilizing CD4+ T cells from *PER2::LUCIFERASE* mice and analyzed rhythmicity in cultured thymic slices as well as purified CD4+ T cells from these animals. Both approaches showed sustained circadian rhythms of luciferase activity indicating the presence of a functional cellular circadian clock. CD40L expression and production of IL-2, IL-4, and IFN-γ by purified CD4+ T cells after polyclonal stimulation followed a circadian rhythm and, for IFN-γ and CD40L, this rhythm was sustained *in vitro* for at least 48 h. Subsequent microarray analysis of *in vitro* cultured and polyclonally stimulated CD4+ T cells showed that the transcription of IκBα is under circadian control which is likely to regulate the activation of NF-κB pathway. In summary, our data strongly indicate that T cells harbor an intrinsic cellular circadian clock and that this clock regulates the IFN-γ and CD40L response following polyclonal stimulation.

Initially we analyzed the light emission of thymic slices from *PER2::LUCIFERASE* reporter mice which contain mostly immature T cells. We showed that cells from the medulla of the thymus, which contains large numbers of T cells, show circadian luciferase activity whereas cells from the cortex do almost not show any luciferase activity. That the thymus in principle is rhythmic had been shown previously by luminescence recordings of whole organ slices [9]. However, our data extends this finding by showing that the rhythmic luciferase expression is almost exclusively due to cells located in the medulla, however, we cannot exclude the involvement of thymic (e.g. stromal) cells using this method. Interestingly, cells from the cortex, which have proliferated, have almost no rhythmic luciferase activity and it had been shown that proliferation desynchronizes the circadian clock of single cells [35]. After this “proof of principle experiment” we switched to the human system where we previously described circadian T cell responses in the presence of APC. First we investigated the circadian expression profile of clock genes in freshly isolated CD4+ T cells. We found rhythmic expression of *Per2*, *Per3*, *E4bp4*, *Rorα*, and *Rev-erbα*. Our findings for *Per2* and *Per3* are in line with a previous report where clock genes were analyzed in freshly isolated PBMCs [13]. Furthermore, we found mRNA expression of IL-2 and IκBα to be under circadian control in unstimulated T cells, suggesting that these genes are partially regulated by a circadian oscillator. Since it is known that the composition - in terms of regulatory, naïve, and memory CD4+ T cells - changes over the circadian cycle [27], [36], [37], the rhythm in clock gene expression could reflect a different pattern in CD4+ T cell subpopulations. It is also known that the cellular circadian clock function is sustained in culture fibroblasts *in vitro* [4], therefore CD4+ T cells were isolated at 6 PM, cultured for 24 h and clock gene expression was analyzed every 3 h. This experimental setup rules out differences in the composition of CD4+ T cells and systemic cues (e.g. hormones, temperature) as a mechanism of circadian clock gene expression. We found rhythmic expression for *Bmal1* (trend), *Per3*, and *Rev-erbα*. These results fit with previous findings showing that the circadian clock in peripheral cells is sustained *in vitro* [4], but differs functionally, at least in part, from the circadian clock of the SCN pacemaker [8]. Of note, *Bmal1* in this experimental setting was rhythmic, which was not the case in the CD4+ T cells which were isolated around the clock. A possible explanation might be different expression of *Bmal1* in the different CD4+ T cell sub-populations which change in their composition in the freshly isolated cells but not in the *in vitro* approach. Furthermore, we did not find *E4bp4*, *Rorα*, and *Per2* to be rhythmic in the *in vitro* approach whereas they were rhythmic in the freshly isolated CD4+ T cells. This difference suggests that the transcription of these genes in CD4+ T cells is mainly driven by systemic cues and not by the cellular circadian oscillator. However, for *Per2* we could show, using CD4+ T cells from *PER2::LUCIFERASE* reporter mice, that the protein is also rhythmic *in vitro* which most likely is caused by post-transcriptional modifications. Luciferase reporter systems have been widely used in circadian biology [35], [38] and allow for continuous real-time monitoring of circadian clock oscillations in living cells. The existence of a cellular circadian clock was previously described in cells of the innate immune system [12], [14], [16]–[18], [20], but little is known about cells from the adaptive immune system. Purified primary T cells from *PER2::LUCIFERASE* reporter mice also sustain their circadian rhythm *in vitro*, but show a moderate variance in period length whereas the phase remains constant. The varying period length could be caused by T cells which are entering the cell cycle, as it has been previously shown that the cell cycle influences the phase and period length of the cellular clocks [35]. Furthermore, we established a *Bmal1-Luc* Jurkat reporter T cell line, but even though we were able to induce circadian rhythms in these cells the reproducibility in terms of phase, period length and amplitude was poor making this cell line not suitable for a circadian *in vitro* approach (data not shown).

Addressing the question whether the previously described circadian/diurnal rhythm in T cell proliferation and cytokine production [26], [27] is dependent on an intrinsic circadian clock in T cells, we showed that circadian T cell activity rhythms are sustained in freshly isolated and purified CD4+ T cells which were polyclonally stimulated *ex vivo*. By this we could exclude that the previously described T cell activity rhythm originates from APCs or serum factors in the culture medium. We found a profound and uniform diurnal rhythm of CD40L expression, IL-2, IL-4, and IFN-γ production in PMA/ionomycin stimulated CD4+ T cells which had a 3–6 h phase advance compared to previously published data [26], [27], [36]. These differences are most likely due to differences of the applied *in vitro* assays. Previous reports used T cell/monocyte co-cultures or whole blood, whereas we have stimulated purified CD4+ T cells. Furthermore, the stimulation agent and/or the time of stimulation differed. Interestingly, the expression of IL-4 is highest approximately 3 h after the peak of *E4bp4* (*Nfil3*) expression which has recently been discovered to be a positive regulator of IL-4 [39]. Furthermore, it was recently shown that E4BP4 is also essential for the production of IL-10 [40], which we have previously shown to be expressed in a circadian fashion after stimulation [26]. However, in this experimental setting we cannot distinguish between effects of the cellular circadian clock and differences in the composition of CD4+ T cells or systemic cues with a circadian rhythm such as cortisol, prolactin etc. which were previously described to influence T cell function by priming the cells *in vivo* [26]. To exclude that systemic cues or changes in CD4+ T cell composition underlie the observed circadian rhythm of T cell activity we cultured freshly isolated CD4+ T cells in serum-free medium for 48 h, detecting a robust circadian rhythm of IFN-γ production and CD40L expression with a dampening of the amplitude in the second 24 h. It can be assumed that this dampening effect was caused by rapid desynchronization of cells due to a lack of coupling and cell differentiation effects. Similar characteristics have been observed in other primary cell systems [35]. In our analysis we focused on genes which were expressed in phase or antiphasic to the level of the IFN-γ response. By choosing this procedure we hoped to exclude *in vitro* artifacts which most likely would not follow a rhythmic expression pattern. The statistically identified candidate genes were analyzed for their biological function. *SGMS2*, previously described to regulate the NF-κB pathway [33], as well as *IκBα* mRNA (an inhibitor of NF-κB) were validated over the complete 48 h experimental period by qPCR. The transcription of *IκBα* is mainly regulated by NF-κB [34] and was therefore analyzed even though it was not identified by the microarray approach. The phase of IκBα does not suggest that it is contributing to the circadian T cell activation, but is rather another indicator of the circadian NF-κB transcriptional activity. Our results are supported by a previous study showing that in freshly isolated non-stimulated peritoneal mouse macrophages *IκBα* is under circadian control [14]. Interestingly, it has been shown that RORα [41], besides NF-κB, is regulating *IκBα* transcription. *Rorα* is part of the circadian clock and we found it to be rhythmically expressed in freshly isolated CD4+ T cells. Furthermore, it has been shown that *E4bp4*, which we found rhythmic in CD4+ T cells, regulates the expression of AP1 (*c-Fos*, *c-Jun*) which also contributes to T cell activation [39]. As it stands, the circadian immune response of CD4+ T cells could be driven by several transcription factors, however likely candidates are NF-κB, AP1, E4BP4, and RORα. Interestingly, we did not find rhythmic mRNA expression of IFN-γ and CD40L mRNA in the microarray, whereas both proteins were rhythmic in stimulated T cells. This might be explained by different translation rates. It has been shown that mRNA translation of abundant proteins is 100 times more efficient than that of low abundant proteins [42]. Hence, if there is a circadian change in the translation rate it could fully explain the circadian rhythm of IFN-γ and CD40L protein. The fact that we did not detect rhythmic clock gene expression in the microarray of stimulated CD4+ T cells is likely due to a repression of clock genes upon stimulation, as previously described [22]. Furthermore, several of the other identified genes which are in phase with the IFN-γ production are interesting such as IFNA7 (interferon alpha7), KLRC2 (killer cell lectin-like receptor subfamily C, member 2) and APOBEC3H (apolipoprotein B mRNA editing enzyme, catalytic polypeptide-like 3H which encodes the cytidine deaminase). IFNA7 is activated via the Jak-Stat pathway which itself is activated by several mediators including IFN-γ, IL-2, and IL-10, all of which we have found to be rhythmic in this or previous studies [26]. Hence, it is possible that the rhythm of IFNA7 mRNA expression is due to early e.g. IFN-γ release and subsequent activation of the Jak-Stat pathway. The rhythmic expression of KLRC2 could indicate a circadian rhythm of T helper 1 responses since KLRC2 has been described to be expressed by T helper 1 but not T helper 2 cells [43]. The circadian rhythm of APOBEC3H, a protein with antiretroviral activity [44], suggests that also antiviral responses could be under circadian control.

In conclusion, we demonstrate using multiple methods that CD4+ T cells harbor a functional circadian oscillator, and show circadian rhythms of IFN-γ and CD40L responses. Our array data suggest that the oscillator might regulate immune function via circadian control of the NF-κB pathway. To date, the potential impact of circadian rhythms is largely ignored in vaccination strategies and in immunosuppressive therapy regiments of autoimmune and allergic diseases because little is known about the circadian rhythm of the adaptive immune system. Our study provides for the first time mechanistic insights into the circadian rhythm of the adaptive immune system by investigating CD4+ T helper cells. Furthermore, we established *in vitro* systems for the analysis of the circadian adaptive immune responses, providing valuable tools for testing the circadian response curve of immunosuppressive drugs on human CD4+ T cells *in vitro*.

## Supporting Information

Figure S1 **Circadian rhythm controls of the subjects.** To control that the analyzed subjects have a normal chronotype we analyzed several established circadian parameters. Blood was sampled from subjects in three hours intervals starting at 6 PM over a 24 h period. The serum/plasma levels of adrenalin, cortisol, melatonin, and prolactin were analysed. Furthermore, we controlled the heart rate and core body temperature (CBT). The p-values depicted in each graph were calculated by Cosinor analysis (Table. S2).
(TIF)

Video S1 **Video of** ***PER2::LUCIFERASE*** **thymic slices.** Thymic slices of male *PER2::LUCIFERASE* mice were cultured in medium supplemented with luciferin and bioluminescence was continuously measured in one hour intervals over approximately 5 days. For histological orientation in the thymic slice see Fig. 1B.
(AVI)

Table S1 **Primer and universal probe number for quantitative PCR.**
(DOC)

Table S2 **The columns “p-value”, “acrophase” and “amplitude” show the results after a cosinor analysis of all donors.** The two columns “single donor analysis” show whether a sinus curve could be fitted significant to the data of each individual donor. The first number shows how many donors showed a significant rhythm and the second number shows how many donors were analyzed.
(DOC)
