# Supplementary figures and images for: Circadian Clocks in Mouse and Human CD4+ T Cells
Source: PLoS One. 2011 Dec 28;6(12):e29801. doi: 10.1371/journal.pone.0029801 (PMC3247291; doi:10.1371/journal.pone.0029801)

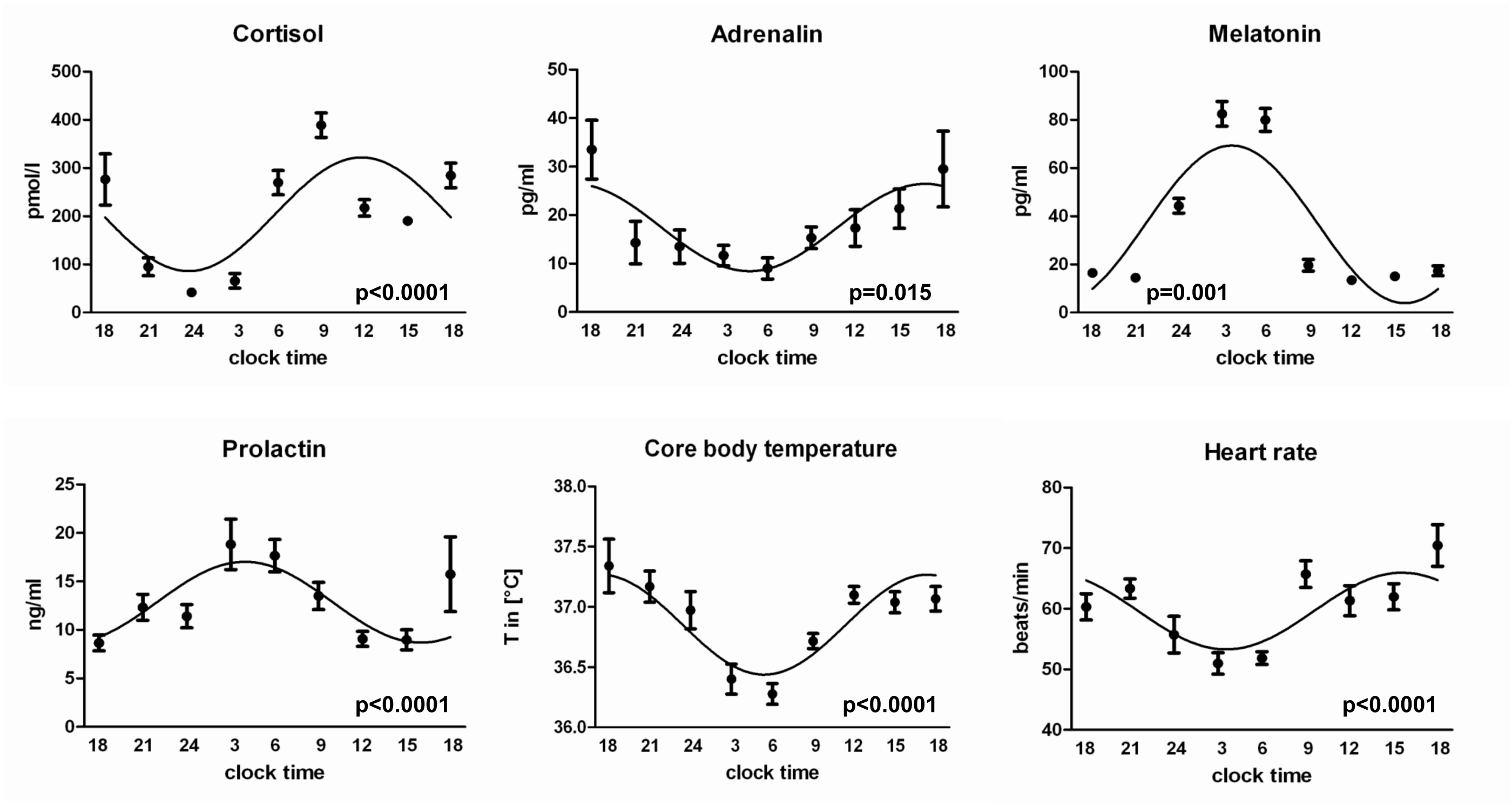

Supplement: Figure S1 — Circadian rhythm controls of the subjects. To control that the analyzed subjects have a normal chronotype we analyzed several established circadian parameters. Blood was sampled from subjects in three hours intervals starting at 6 PM over a 24 h period. The serum/plasma levels of adrenalin, cortisol, melatonin, and prolactin were analysed. Furthermore, we controlled the heart rate and core body temperature (CBT). The p-values depicted in each graph were calculated by Cosinor analysis (Table. S2). (TIF) [file pone.0029801.s001.tif]
