# Supplementary material for: Circadian Clocks in Mouse and Human CD4+ T Cells
Source: PLoS One. 2011 Dec 28;6(12):e29801. doi: 10.1371/journal.pone.0029801 (PMC3247291; doi:10.1371/journal.pone.0029801)
Supplement: Table S1 — Primer and universal probe number for quantitative PCR. (DOC) [file pone.0029801.s003.doc]

**Tab. S1: Primer and universal probe number for quantitative PCR**:

| **Table S1** | **Primer sequence (5'→3)** | **UPL probe no.** |
| --- | --- | --- |
| **ALAS-For** | CCC TCT TCA CCC TGG CTA A | **64** |
| **ALAS-Rev** | AGG CAT GGT TCC CAG AAT C |  |
| **B2M-For** | TTC TGG CCT GGA GGC TAT C | **42** |
| **B2M-Rev** | TCA GGA AAT TTG ACT TTC CAT TC |  |
| **BMAL1-For** | GCA GCT CCA CTG ACT ACC ATT | **19** |
| **BMAL1-Rev** | GCA TCT CCT AGT GGG CAT CT |  |
| **CD40L-For** | TGT ATC TTC ATA GAA GGT TGG ACA AG | **2** |
| **CD40L-Rev** | CCT CAC AGT TCA GTA AGG ATA AGG A |  |
| **Clock-For** | GAG AGC GCG AAG GAA ATC T | **70** |
| **Clock-Rev** | GCA GCT TTG CAG GAA CAA GTA |  |
| **Cry1-For** | CTA CTC ACC GCC AGA GCA A | **19** |
| **Cry1-Rev** | GCT ATG CCA TCG TAG AAT TGG |  |
| **Cry2-For** | GAA CCA CGA CGA GAC CTA CG | **17** |
| **Cry2-Rev** | CAA GTC CTT CAG TGG GGA AC |  |
| **Dbp-For** | CTA AAC CTC CGC TCT CGG TA | **4** |
| **Dbp-Rev** | CGC GGT CCT AAA GAT GGT T |  |
| **E4BP4-For** | CCC CTT TCT TTC TCC TCG TC | **15** |
| **E4BP4-Rev** | AGT TGG GCC TCC TTC GTT AT |  |
| **G6PDH-For** | GCA AAC AGA GTG AGC CCT TC | **82** |
| **G6PDH-Rev** | GGG CAA AGA AGT CCT CCA G |  |
| **HPRT-For** | TGA CCT TGA TTT ATT TTG CAT ACC | **73** |
| **HPRT-Rev** | CGA GCA AGA CGT TCA GTC CT |  |
| **IFN-g-For** | GGC ATT TTG AAG AAT TGG AAA G | **21** |
| **IFN-g-Rev** | TTT GGA TGC TCT GGT CAT CTT |  |
| **IkBa-For** | TGT GCC TCT TCT AGC AAT GGA | **25** |
| **IkBa-Rev** | TTC TGG TTT GTT GAG CAG CTT |  |
| **IL-2-For** | AAG TTT TAC ATG CCC AAG AAG G | **65** |
| **IL2-Rev** | AAG TGA AAG TTT TTG CTT TGA GCT A |  |
| **PBGD-For** | CCT GTT TAC CAA GGA GCT TGA | **37** |
| **PBGD-Rev** | GGA GTG AAC AAC CAG GTC CA |  |
| **Per2-For2** | GGA GTT CAA GGC TAC GGT GA | **19** |
| **Per2-Rev2** | CCA GTG GCC CAC ACA TTT |  |
| **Per3-For** | TCC AGT TGG TCC AGC TTT G | **1** |
| **Per3-Rev** | TCC TCA TTT AGT GGG CTC GT |  |
| **Rev-ERBA-For** | AAC TCC CTG GCG CTT ACC | **17** |
| **Rev-ERBA-Rev** | GAA GCG GAA TTC TCC ATG C |  |
| **RORA-For** | GCA TTA TTT TCT GCA TTT GTA CTG A | **46** |
| **RORA-Rev** | TGC AGT TTT TCA ATT TTT ACC TTT TC |  |
| **SGMS2 For** | CTA CCT GTG CCT GGA ATG C | **47** |
| **SGMS2 Rev** | CGT TGA ACT TTT GCC TGA GA |  |
