# Supplementary material for: Circadian Clocks in Mouse and Human CD4+ T Cells
Source: PLoS One. 2011 Dec 28;6(12):e29801. doi: 10.1371/journal.pone.0029801 (PMC3247291; doi:10.1371/journal.pone.0029801)
Supplement: Table S2 — The columns “p-value”, “acrophase” and “amplitude” show the results after a cosinor analysis of all donors. The two columns “single donor analysis” show whether a sinus curve could be fitted significant to the data of each individual donor. The first number shows how many donors showed a significant rhythm and the second number shows how many donors were analyzed. (DOC) [file pone.0029801.s004.doc]

**Tab. S2: Statistical values, amplitudes and acrophases of all parameters as well as single donor analysis sorted by figures:**

| **Table S2** | **Figure** | **p-value** | **Acro-phase (in [h])** | **Amplitude** | **Single donor analysis**  **(p<0.1)** | **Single donor analysis**  **(p<0.05)** |
| --- | --- | --- | --- | --- | --- | --- |
| CD40L (% ) | 1 | 0.0017 | 18:51 | 57.37 | 2/7 | 2/7 |
| CD40L (MFI) | 1 | 0.0125 | 18:31 | 25.03 | 4/7 | 2/7 |
| IFN-γ+ CD40L+ (%) | 1 | 0.0031 | 19:05 | 65.25 | 3/7 | 3/7 |
| IFN-γ (MFI) | 1 | <0.00001 | 19:54 | 43.82 | 5/7 | 3/7 |
| IL-2 (%) | 1 | 0.027 | 18:05 | 43.01 | 3/7 | 2/7 |
| IL-2 (MFI) | 1 | 0.0062 | 18:12 | 15.63 | 1/7 | 0/7 |
| IL-4 (%) | 1 | 0.0003 | 18:15 | 67.71 | 3/7 | 2/7 |
| IL-4 (MFI) | 1 | 0.22 | 16:40 | 12.52 | 2/7 | 2/7 |
| IL-17 (%) | 1 | 0.11 | 19:19 | 44.71 | 3/7 | 3/7 |
| IL-17 (MFI) | 1 | 0.14 | 19:02 | 15.51 | 0/7 | 0/7 |
| *Bmal1* (mRNA) | 2 | 0.1116 | 22:07 | 6.59 | 2/7 | 2/7 |
| CD40L (mRNA) | 2 | 0.16 | 08:07 | 5.59 | 2/7 | 1/7 |
| *Clock* (mRNA) | 2 | 0.58 | 04:41 | 2.32 | 0/7 | 0/7 |
| *Cry1* (mRNA) | 2 | 0.25 | 04:09 | 5.57 | 2/7 | 1/7 |
| *Cry2* (mRNA) | 2 | 0.059 | 13:28 | 11.86 | 2/7 | 1/7 |
| *Dbp* (mRNA) | 2 | 0.22 | 05:17 | 5.72 | 1/7 | 1/7 |
| *E4bp4* (mRNA) | 2 | 0.044 | 14:00 | 17.98 | 1/7 | 0/7 |
| IFN-γ (mRNA) | 2 | 0.83 | 05:35 | 2.92 | 1/7 | 1/7 |
| IL-2 (mRNA) | 2 | 0.024 | 11:32 | 12.05 | 1/7 | 1/7 |
| IκBα (mRNA) | 2 | 0.097 | 11:24 | 6.62 | 1/7 | 0/7 |
| Per2 (mRNA) | 2 | 0.017 | 05:03 | 9.59 | 2/7 | 1/7 |
| Per3 (mRNA) | 2 | <0.00001 | 06:08 | 22.59 | 6/7 | 5/7 |
| Rev-erbα (mRNA) | 2 | <0.00001 | 01:35 | 19.91 | 5/7 | 5/7 |
| Rorα (mRNA) | 2 | 0.02 | 06:39 | 9.27 | 1/7 | 1/7 |
| *Bmal1* (mRNA) | 3 | 0.0515 | 09:08 | 14.3 | 0/3 | 0/3 |
| *CD40L* (mRNA) | 3 | 0.11 | 23:33 | 7.97 | 1/3 | 1/3 |
| *Clock* (mRNA) | 3 | 0.55 | 17:43 | 8.01 | 0/3 | 0/3 |
| *Cry1* (mRNA) | 3 | 0.43 | 13:00 | 16.04 | 0/3 | 0/3 |
| *Cry2* (mRNA) | 3 | 0.12 | 17:13 | 21.91 | 1/3 | 1/3 |
| *Dbp* (mRNA) | 3 | 0.12 | 10:57 | 32.73 | 1/3 | 0/3 |
| *E4bp4* (mRNA) | 3 | 0.89 | 15:03 | 8.49 | 0/3 | 0/3 |
| IFN-γ (mRNA) | 3 | 0.23 | 11:49 | 27.44 | 1/3 | 0/3 |
| Per2 (mRNA) | 3 | 0.43 | 14:48 | 21.13 | 0/3 | 0/3 |
| Per3 (mRNA) | 3 | 0.0001 | 15:37 | 32.76 | 3/3 | 2/3 |
| Rev-erbα (mRNA) | 3 | 0.046 | 21:17 | 41.74 | 0/3 | 0/3 |
| Rora (mRNA) | 3 | 0.67 | 14:29 | 9.62 | 0/3 | 0/3 |
| CD40L (%), 0-24 h | 4 | 0.017 | 5:07 | 56.86 | 3/5 | 1/5 |
| CD40L (%), 24-48 h | 4 | 0.59 | 6:27 | 18.36 | 0/5 | 0/5 |
| CD40L (MFI), 0-24 h | 4 | 0.41 | 4:57 | 21.57 | 2/5 | 1/5 |
| CD40L (MFI), 24-48 h | 4 | 0.78 | 8:21 | 11.15 | 0/5 | 0/5 |
| IFN-γ+CD40L+ (%), 0-24 h | 4 | 0.037 | 5:24 | 70.1 | 2/5 | 1/5 |
| IFN-γ+CD40L+ (%), 24-48 h | 4 | 0.36 | 5:32 | 43.05 | 0/5 | 0/5 |
| IFN-γ (MFI), 0-24 h | 4 | 0.048 | 5:24 | 32.65 | 2/5 | 2/5 |
| IFN-γ (MFI), 24-48 h | 4 | 0.59 | 8:38 | 8.74 | 0/5 | 0/5 |
| IκBα (mRNA), 0-24 h | 5 | 0.0031 | 5:50 | 20.82 | 2/3 | 1/3 |
| IκBα (mRNA), 24-48 h | 5 | 0.02 | 22:48 | 12.75 | 2/3 | 1/3 |
| SGMS2 (mRNA), 0-24 h | 5 | 0.055 | 1:47 | 50.98 | 1/3 | 1/3 |
| SGMS2 (mRNA), 24-48 h | 5 | 0.39 | 14:10 | 11.82 | 2/3 | 1/3 |
| Adrenalin | Suppl.-Fig.1 | 0.0003 | 16:46 | 42.8 | 3/7 | 3/7 |
| Core body temperature | Suppl.-Fig.1 | <0.00001 | 17:23 | 1.12 | 6/7 | 6/7 |
| Cortisol | Suppl.-Fig.1 | <0.00001 | 11:45 | 58.47 | 4/7 | 4/7 |
| Heart rate | Suppl.-Fig.1 | <0.00001 | 15:33 | 10.46 | 4/7 | 2/7 |
| Melatonin | Suppl.-Fig.1 | <0.00001 | 03:40 | 96.92 | 7/7 | 7/7 |
| Prolactin | Suppl.-Fig.1 | <0.00001 | 03:40 | 27.56 | 5/7 | 3/7 |
